# Habitat suitability modeling and conservation status of *Salvadora oleoides* and *Tamarix aphylla* in tropical thorn forest

**DOI:** 10.1371/journal.pone.0306570

**Published:** 2024-12-31

**Authors:** Majid Hussain, Hassan Akhtar, Zafeer Saqib, Muhammad Tayyab Khan, Zarak Khan Afridi, Hasnain Afzal, Ume Habiba, Sangam Khalil, Ghulam Raza, Hamid Ur Rahman, Sher Shah, Muhammad Sohail Yousaf, Tahani Awad Alahmadi

**Affiliations:** 1 Department of Forestry and Wildlife Management, The University of Haripur, Haripur, Khyber Pakhtunkhwa, Pakistan; 2 Department of Environmental Sciences, International Islamic University, Islamabad, Pakistan; 3 Department of Land, Environment, Agriculture and Forestry, University of Padova, Legnaro, Italy; 4 Institute of Forest Sciences, Faculty of Agriculture and Environment Sciences, The Islamia University Bahawalpur, Bahawalpur, Pakistan; 5 Department of Biological Sciences, University of Baltistan, Skardu, Pakistan; 6 Institute of Agriculture Sciences and Forestry, University of Swat, Swat, Khyber Pakhtunkhwa, Pakistan; 7 Yale School of the Environment, New Haven, Connecticut, United States of America; 8 Department of Pediatrics, College of Medicine and King Khalid University Hospital, King Saud University, Medical City, Riyadh, Saudi Arabia; Central South University of Forestry and Technology, CHINA

## Abstract

The habitat suitability of *Salvadora oleoides* and *Tamarix aphylla* can be one of the most significant steps towards conserving these tree species. Habitat loss presents a critical threat to the existence of *S*. *oleoides* and *T*. *aphylla*. Protecting their suitable habitats and implementing conservation approaches is crucial to address this challenge. By ensuring the preservation of their habitats and adopting effective conservation strategies, we can mitigate the threat of habitat loss and promote the survival of these species. The potential distribution of *S*. *oleoides* and *T*. *aphylla* was predicted using a MaxEnt model. This study also presents the conservation status of *S*. *oleoides* and *T*. *aphylla* in the tropical thorn forests of the Bahawalpur subdivision. Data were gathered from the field survey based on bioclimatic variables. Overall, 20 sample plots were taken, and the coordinates were recorded for each sample plot. MaxEnt software and the environmental variables were used to study each tree species separately (19 bioclimatic variables were used). The Jackknife test was conducted to find the total general tree cover and mean temperature. The MaxEnt model showed high accuracy for each tree species, with the receiver operating characteristics (ROC) area under the curve (AUC) training mean testing values for *S*. *oleoides* being 0.976 and *T*. *aphylla* 0.987. The study showed that both species were distributed irregularly in the tropical thorn forest of the Bahawalpur subdivision. The results highlight that it is essential to implement proven long-term management and conservation techniques to ensure the well-being and sustainability of forest trees in the Bahawalpur sub-division. In conclusion, concerted efforts to map, understand habitat suitability, and raise awareness of endangered species in the tropical thorn forest are crucial for effective conservation planning and resource allocation in the face of climate change.

## 1. Introduction

Tropical thorn forests, which occupy only 10% of the Earth’s surface, are home to approximately half of all plant species [1]. Despite this, the tropical thorn forest in Pakistan has not received much attention from the scientific and botanical communities. This neglect has led to a lack of representatives who can emphasize their importance and advocate for their restoration and conservation [2]. Although the government recognizes the urgent need to expand forest cover in Pakistan, few tangible actions have been taken since 1955 to address this problem effectively [3]. However, the remaining forest resources in Pakistan are very vulnerable and at risk.

The Punjab plains in Pakistan once were engulfed with the beauty of natural tropical thorn forests [4]. *Salvadora oleoides* is an evergreen tree species found in Pakistan’s tropical forests that is of great ecological and ethnobotanical importance. The decrease in the population of Indigenous plants can be attributed to various factors, such as habitat loss, tree cutting, soil loss, overspending, and the introduction of non-indigenous species [5]. As a result, Pakistan is now considered one of the poorest countries in the world in terms of the expected loss of natural resources over the next three decades [6]. The tropical thorn forest is sustained through natural seed regeneration, as well as vegetative methods such as root sucking and harvesting. *S*. *oleoides* can reproduce by natural overlap [7].

Species distribution models (SDM) are well-established techniques that implement various methods to integrate data on species occurrences and preferences with environmental niche components to find the suitable habitat of a species. For example, SDMs can be used to model the distribution of endangered plant species by analyzing data on bioclimatic variables such as temperature, precipitation, and land cover [8]. Many SDMs (such as MaxEnt, BIOCLIM, and Climax) have been developed to predict the species distribution. The models have become increasingly crucial for understanding the spatial patterns of biodiversity and the potential impacts of environmental change on species distributions [9].

Habitat suitability models (HSM) are often considered practical implementations of the concept of ecological niche, which involves the use of environmental factors to forecast the absence or abundance of a species in a research area [10]. HSM and the Geographic Information System (GIS) are two robust instruments that have significantly improved the domain of ecology, especially in the realms of conservation and resource exploitation. These tools have allowed researchers to generate more accurate predictions of ecological patterns and processes and better understand the complex relationships between species and their environments [11]. As stated by [12, 13], MaxEnt, a widely recognized SDM, focuses primarily on presence modeling. This approach involves maximum entropy modeling and has been proven to be successful in predicting the distributions of different species of flora and fauna [14]. Through GIS, it is possible to combine geographic distribution data of various species, allowing the determination of the location of critical areas for conservation efforts [15, 16].

*S*. *oleoides* is native to Pakistan and can be found throughout the Punjab plains province. It is typically found in hot and arid areas of Punjab. It belongs to the Salvadoraceae family (which has 12 species and 3 genera) and is commonly known as Bada Pilu or Vridhpilu in Pakistan [17–19]. This species is known for tolerating arid environments [18], including areas with mean rainfall below 200 mm [19–22]. Locals have noted this tree’s dried fruits as improving bovine milk’s productivity. The vast root system of this species helps prevent soil erosion, while the thick canopy of mature trees acts as a windbreak. Roots and branches of *S*. *oleoides* have traditionally been used as toothpicks and toothbrushes [21].

*Tamarix aphylla* is another dominant species found in tropical thorn forests. It is native to the Middle East, including Pakistan, Central Asia, North Africa, and Arabia. It is common in the Punjab, Sindh, Baluchistan, and KPK plains of Pakistan [22–24]. In addition, it is widely cultivated in the dunes of the Thal and Cholistan deserts. *T*. *aphylla* is an evergreen and fast-growing tree with several economic and commercial advantages. This tree is highly resistant to wet and saline soils of arid or desert areas) and saline soils, making it the most adaptable species to these conditions [24]. Its barks and galls have medicinal properties, and its branches are cut and used for basketry production. This plant can thrive in harsh environmental conditions and requires little maintenance [24, 25]. This study aims to identify the distribution and suitable habitats of *S*. *oleoides* and *T*. *aphylla* within the tropical thorn forest of the Bahawalpur subdivision. Additionally, it seeks to assess the conservation status of these species in the specified region.

## 2. Materials and methods

### 2.1 Study area

**Fig 1:**
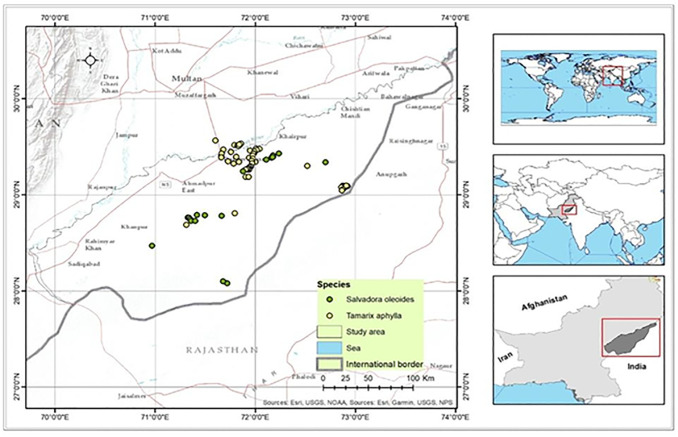
Map of the study area showing the distribution of two species of native trees.

This research was carried out in the Bahawalpur subdivision of Punjab, Pakistan Bahawalpur is located at (29° 24′0′′N, 71° 41′0′′E) and covers an area of 24,830 km^2^ (Fig 1). The Tropical Thorn Forest in the Bahawalpur subdivision has an area of 83,140,586.14 m^2^. The main source of water supplies is a river and a well-irrigated canal system, with annual precipitation ranging from 90 to 200 millimeters. This region is located in the southeast part of Punjab and experiences hot summers, sandstorms in May and June, and cold winters. The tropical plains thorn forest is the natural vegetation that covers the area [26]. Common species in this region include *T*. *aphylla*, *S*. *oleoides*, *Prosopis cineraria*, *Prosopis spicigera*, *Acacia nilotica*, and *Acacia leucophloea*. The map is prepared using ArcGIS 10.5 software. The three habitats in this area are tropical thorn forests, dune scrub, and irrigated plantations. In February 1978, tropical thorn forests and irrigated plantations showed the highest levels of cover, while the Calligonum scrub type had a cover ranging from 0 to 70%. Each habitat offers sufficient escape cover, ranging from 20% to 100%.

### 2.2 Field surveys

Field surveys were conducted to collect data in the research area in February 2023, and the target species were forecasted to be present or absent. Data were collected randomly in the study area. Twenty sample plots (0.01% of the total study area) were evaluated in the Bahawalpur subdivision [27]. All tree species were identified, and their total number was noted. Geographic coordinates and elevation measurements were also recorded for each tree in each plot. Each species was evaluated in the field for its historical distribution range, frequency, and extension [28].

### 2.3 Development of the model and species occurrence data

The occurrence and climatic data of the species were gathered to create models of habitat suitability or species distribution for both species. Field surveys were carried out within the Bahawalpur Tropical Thorn Forest, with meticulous attention devoted to acquiring data on the *S*.*oleoides* and *T*. *aphylla* species. The data collection process involved the compilation of locality points meticulously collected from the field, ensuring a comprehensive documentation of the occurrence of the species.

### 2.4 Climatic data

Climate data was obtained from the WorldClim database and the Lahore Metrological Centre for the Bahawalpur station [28]. These factors are used in the modeling of species distribution [28]. Given that the environmental layers obtained covered the entire global landscape, it became imperative to use the clipping technique to isolate and extract specific satellite imagery related to the geographical region of Pakistan from within this extensive and comprehensive dataset.

The MaxEnt software needs three different folders for a single species. These folders are divided into ambient layers, existing data, and output. All environmental layers were resampled, spatial resolution was taken, and they were all saved in the same folder called "environmental variables." To analyze the locality data surveys, we carried out five duplicate runs of MaxEnt, randomly selected predetermined for each run. After conducting five repeated MaxEnt runs, the expected, anticipated distribution matched closely. The complete historical range of the species. We run the MaxEnt software after receiving all the required information, including the current data and the environment layers. MaxEnt model output was evaluated with ArcGIS Edition 10.5 software.

To enter the data for MaxEnt analysis, the following procedure was followed.

1. We only examined the presence data of the selected species in the sample. All environmental layers for tree species were studied in the Bahawalpur subdivision, focusing only on the presence points of the chosen tree species in the sample.
2. There were two types of data: continuous and categorical. We organize all land use, soil vegetation, or forest-type data and choose "categorical" from the environmental layer drop-down option. On the other hand, data related to precipitation, cloud cover, rainfall, humidity, temperature, frost, etc., were considered continuous data.
3. Subsequently, we run the model that produces the prediction images and perform a jackknife analysis to determine the significance of the variables.

### 2.5 IUCN conservation status assessment criteria

The categories and criteria of the International Union for Conservation of Nature (IUCNVersion 3.1 were used to determine the conservation status of plant species. This included evaluating plant species’ extinction threat and awarding them a conservation status based on the criteria provided in the IUCN framework.

### 2.6 Species identification

These species were identified by the Cholistan Institute of Desert Studies Bahawalpur, the Bahawalpur Forest Department, the Department of Forestry (Islamic University of Bahawalpur), and the Department of Forestry and Wildlife Management of the Industrial Forestry Lab of the University of Haripur. This study did not require any permits, as no experiments were conducted on animals, humans, or other living organisms. The data was collected with the help of a questionnaire survey and field sampling, whereas no destructive sampling was applied during the data collection, so there was no need for any permit.

## 3. Results

### 3.1 Dynamics of *Salvadora oleoides*

**Fig 2:**
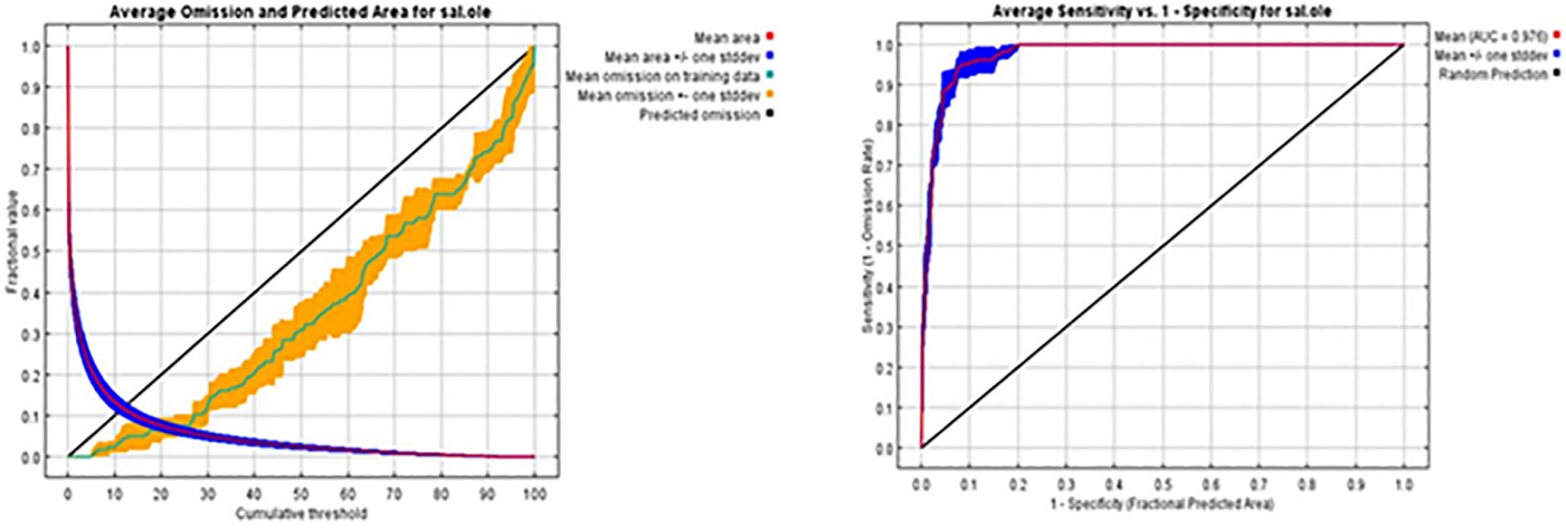
(a) Predicted area and omission rates of *Salvadora oleoides*, and (b) the area under the receiver operating characteristics curve analysis (AUC).

The estimated area and the test omission rate are displayed as functions of the cumulative threshold averaged over numerous runs. The closer the omission on the sample line is to the predicted omission, the more accurate the model developed (Fig 2a). The Area Under the Curve (AUC) of the Receiver Operating Characteristics (ROC) curve was used to evaluate the predictive power of the developed model. The standard deviation for the replicate runs is 0.005, and the average training AUC is 0.976. The ROC curve was averaged over replicate runs. The standard deviation is 0.005. The average training AUC for replicate runs is 0.976 (Fig 2b).

**Fig 3:**
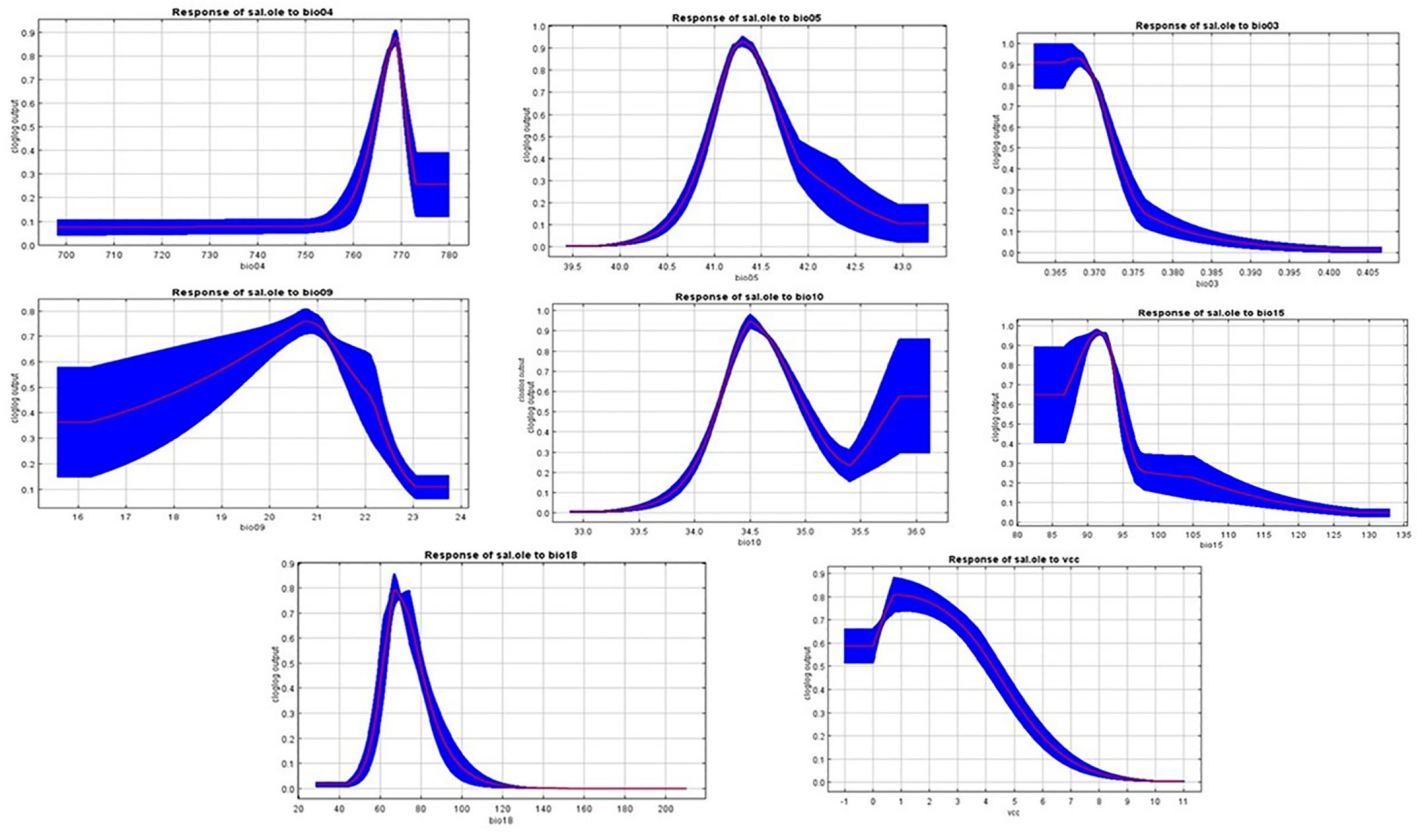
Response curves to each environmental variable for *Salvadora oleoides*.

Response curves typically represent spatial illustrations that show how a specific variable, as indicated in Table 1, changes in response to variations in another variable. The depiction in Fig 3 highlights that these curves are essential tools for visualizing and comprehending the interactions between different variables.

**Table 1:** Shows the variables that contribute significantly to the MAXENT models fitted for *S*. *oleoides*.

Variable	% contribution	Permutation Importance
*Isothermality*	9.7	39.2
*Temperature Seasonality*	13.2	0.2
*Max Temperature of Warmest Month*	0	1.2
*Mean Temperature of Wettest Quarter*	23.2	13.7
*Mean Temperature of Driest Quarter*	0.2	1.6
*Mean Temperature of Warmest Quarter*	2.3	0.2
*Precipitation Seasonality (Coefficient of Variation)*	17.6	0.6
*Precipitation of Warmest Quarter*	20.o	24.3

### 3.2 Dynamics of *Tamarix aphylla*

**Fig 4:**
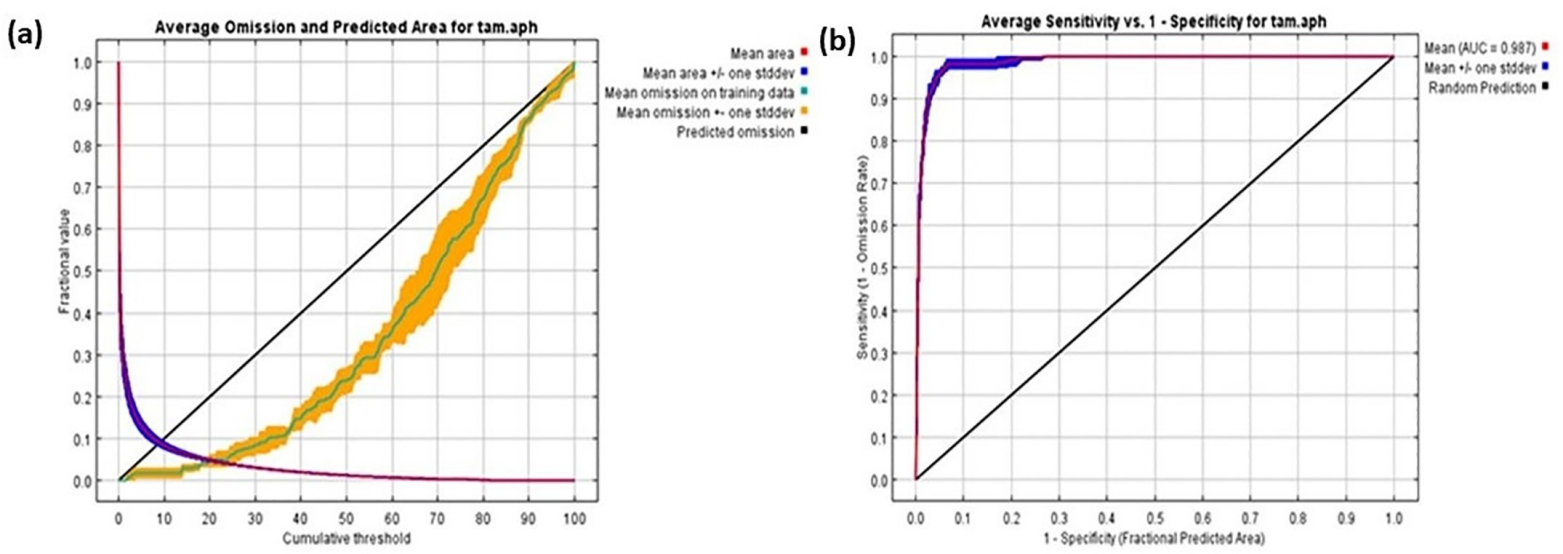
Predicted area and omission rates of *Tamarix aphylla*, and (b) the area under the receiver operating characteristics curve analysis (AUC).

Fig 4a illustrates the rate of training omission and the predicted area as functions of the cumulative threshold, averaged across all replicate runs. This graph demonstrates the model’s accuracy and reliability. The variable’s significance in the table is indicative of its substantial contribution to the MaxEnt model. Table 2 details the relative contributions of different environmental variables to the MaxEnt model’s performance. During each iteration of the training process, the model first estimates the regularized gain and adjusts the contribution of each variable accordingly. If the regularized gain increases, it is added to the variable’s contribution; if the change in the absolute value of Lambda is negative, the contribution is reduced. For the second estimate, each environmental variable is permuted in the training presence and background data, one at a time, to further refine the model’s assessment of each variable’s impact.

**Table 2:** Shows the variables that contribute significantly to the MAXENT models fitted for *Tamarix aphylla*.

Variable	% contribution	Permutation Importance
*Isothermality*	1.7	20.9
*Temperature Seasonality*	6.7	0.6
*Max Temperature of Warmest Month*	7.5	0
*Mean Temperature of Wettest Quarter*	38.4	21
*Mean Temperature of Driest Quarter*	8.7	0.7
*Mean Temperature of Warmest Quarter*	4.1	14.3
*Precipitation Seasonality (Coefficient of Variation)*	4.3	2
*Precipitation of Warmest Quarter*	1.4	23.7

Fig 4b displays the ROC curve, averaged over multiple replicate runs. The graph illustrates the model’s performance, with the AUC serving as a key metric. For these replicate runs, the average training AUC is 0.987, indicating excellent model accuracy. The consistency of the model is further supported by a low standard deviation of 0.003. This high AUC value and low variability highlight the robustness and predictive power of the model across different training iterations.

**Fig 5:**
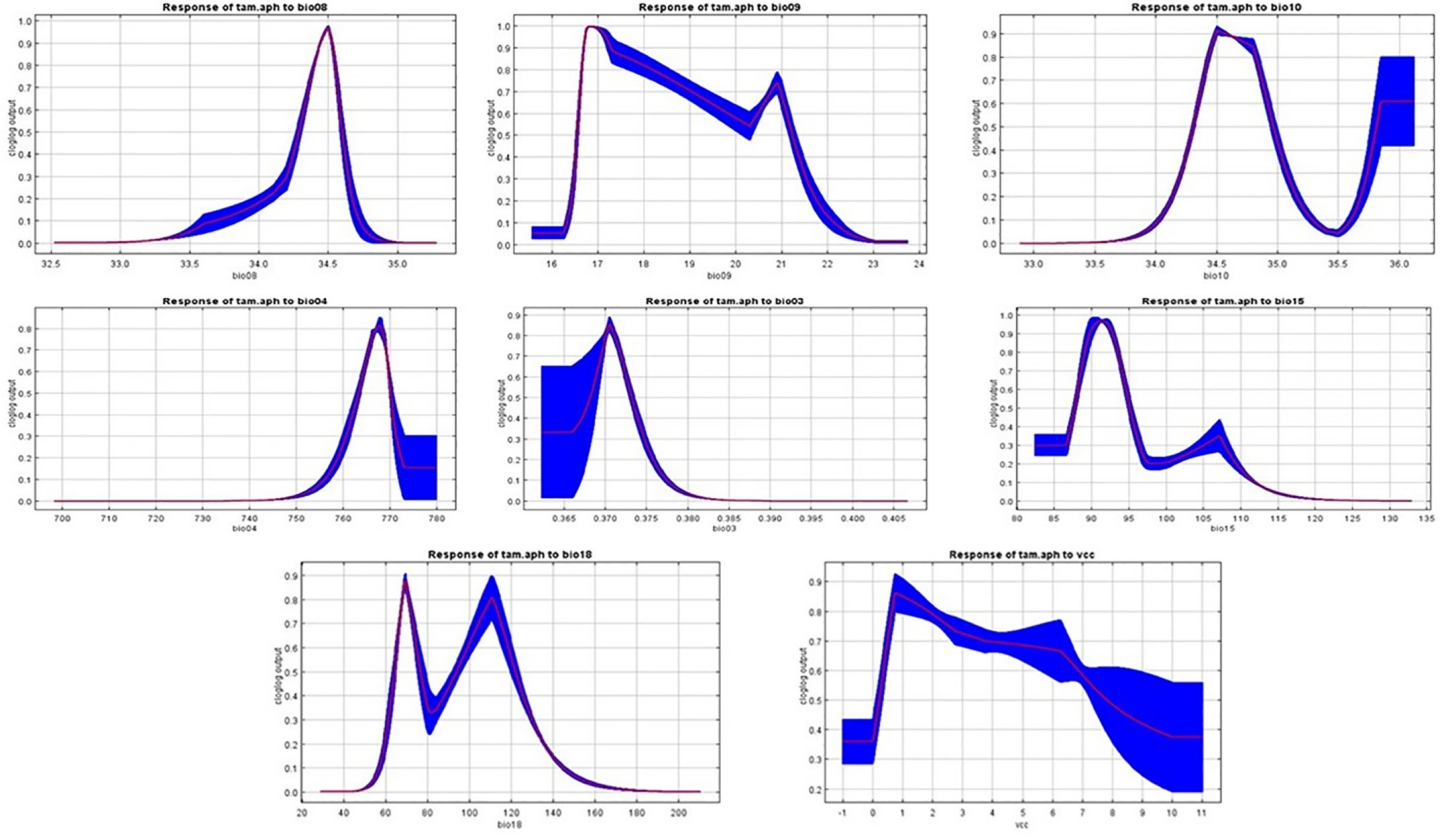
Response curves to each environmental variable for *Tamarix aphylla*.

Response curves are invaluable for visually representing how a specific parameter changes in response to variations in another parameter, often within a spatial context. Fig 5 showcases the significance of these curves by demonstrating their utility in understanding the relationships between different parameters. These curves provide a clear and intuitive way to interpret how alterations in one parameter can influence another, making them crucial for analyzing complex interactions and predicting the effects of changes in environmental or other variable conditions.

### 3.3 Jackknife plot of *Salvadora oleoides* and *Tamarix aphylla*

**Fig 6:**
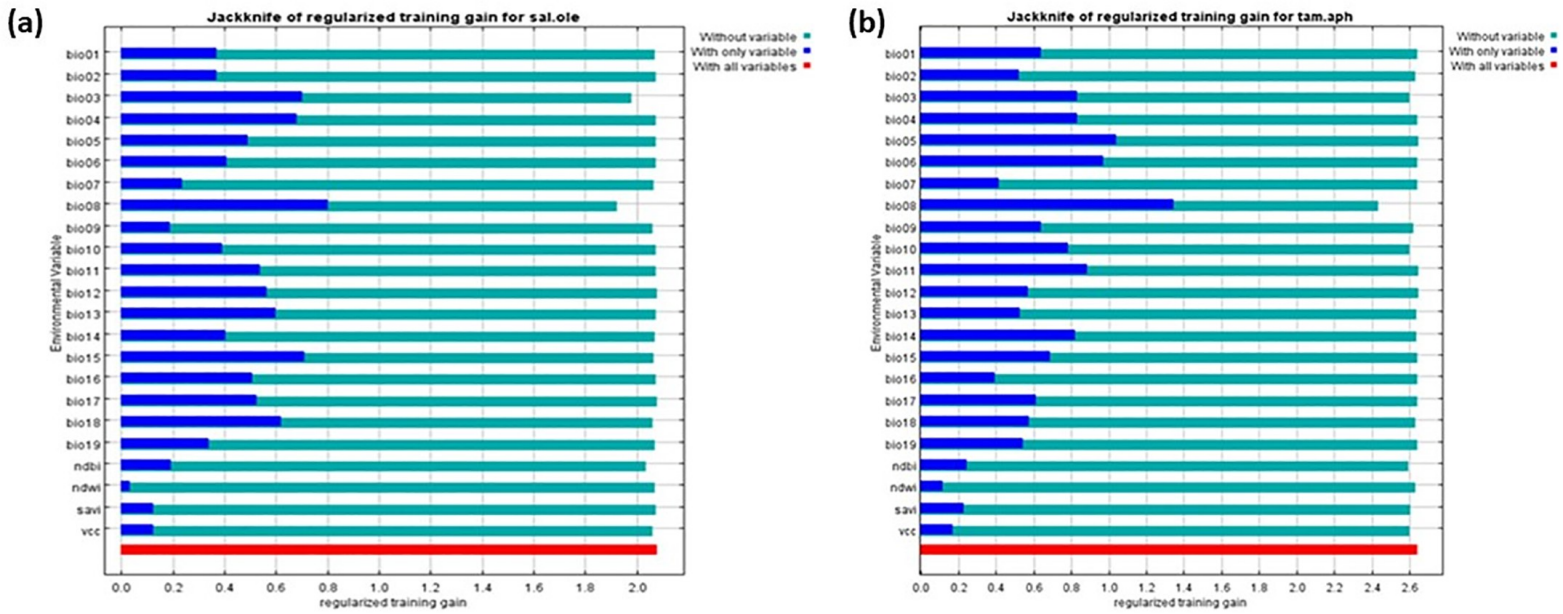
Jackknife plot of training gain for *Salvadora oleoides* (a) and *Tamarix aphylla* (b).

Regarding *S*. *oleoides* Fig 6a presents the results of the Jackknife test for variable importance. The analysis highlights that Bio08 (Mean Temperature of the Wettest Quarter) emerges as the most influential environmental variable. Bio08 yields the highest regularized training gain when used independently among all variables. Additionally, the exclusion of Bio08 leads to the most significant decrease in regularized gain, underscoring its critical role in the model. The values depicted in Fig 6a are averaged across multiple replicate runs, further validating the prominence of Bio08 in the environmental dataset.

Regarding *T*. *aphylla*, Fig 6b shows Bio08 (Mean Temperature of the Wettest Quarter), this analysis’s most influential environmental variable. Bio08 yields the highest gain when used alone, indicating its strong predictive power. Conversely, omitting Bio08 from the model results in the greatest reduction in gain, suggesting that it contains the most critical information. The values displayed are averaged across multiple replicate runs, as shown in Fig 6b, reinforcing the consistent significance of Bio08 in the model.

### 3.4 Habitat suitability *Salvadora oleoides* and *Tamarix Aphylla*

**Fig 7:**
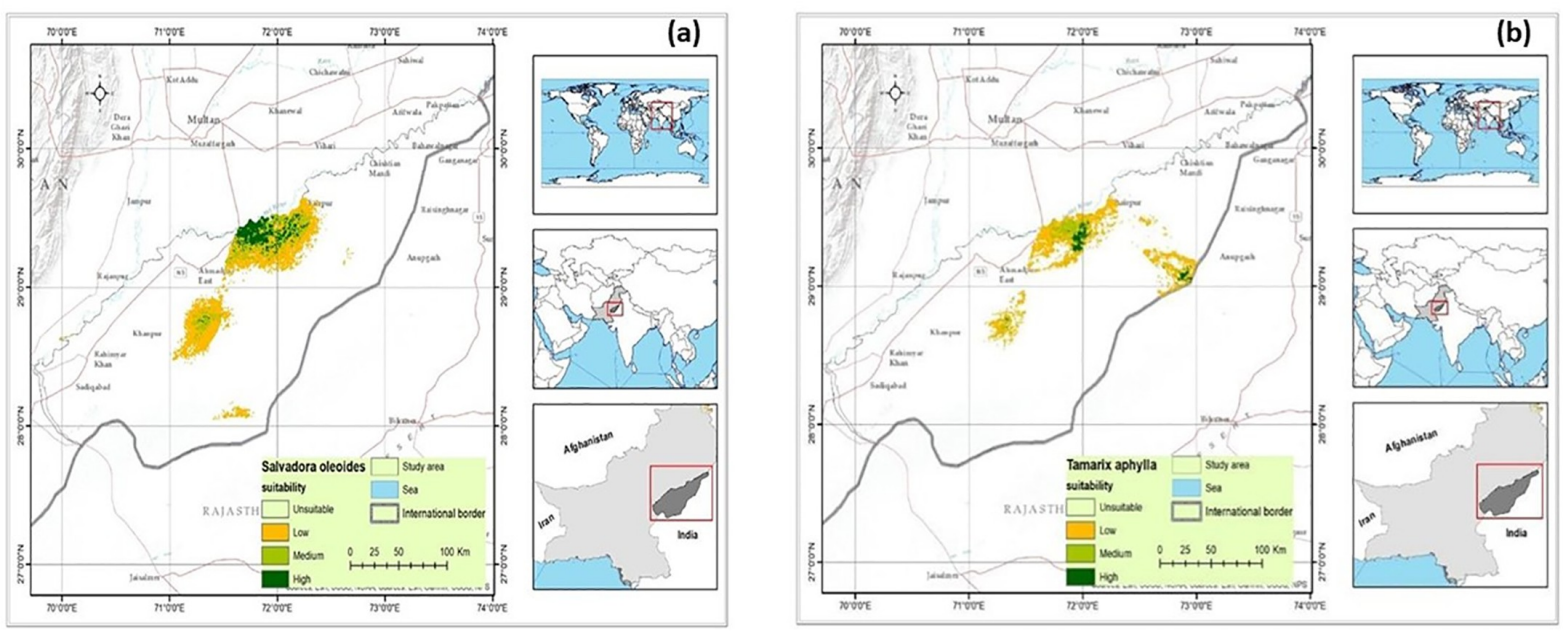
The habitat suitability of *Salvadora oleoides* and *Tamarix aphylla* identified using MaxEnt software. High suitability (60–100%), Moderate suitability (40–60%), Low suitability (20–40%) and Unsuitable habitat (0–20%).

The color gradient indicates the suitability of the habitat, with green representing very highly suitable areas (60–100%), light green indicating moderately suitable regions (40–60%), yellow reflecting less suitable areas (20–40%), and light colors indicating unsuitable habitat (0–20%), as depicted in Fig 7. The Bahawalpur subdivision has identified a population of 51 individual *T*. *Aphylla* trees. However, this population has experienced a concerning 40% decrease, resulting in its classification as vulnerable. Contributing factors to this decline include exploiting the species for fuelwood and medicinal purposes. These findings underscore the importance of implementing conservation measures to mitigate further population reductions and protect the habitats of *S*. *oleoides* and *T*. *Aphylla* within the Bahawalpur subdivision. Efforts to address habitat degradation and regulate the unsustainable harvesting of these species are crucial for their long-term survival (Fig 7).

### 3.5 Conservation status of *Salvadora oleoides* and *Tamarix Aphylla*

**Fig 8:**
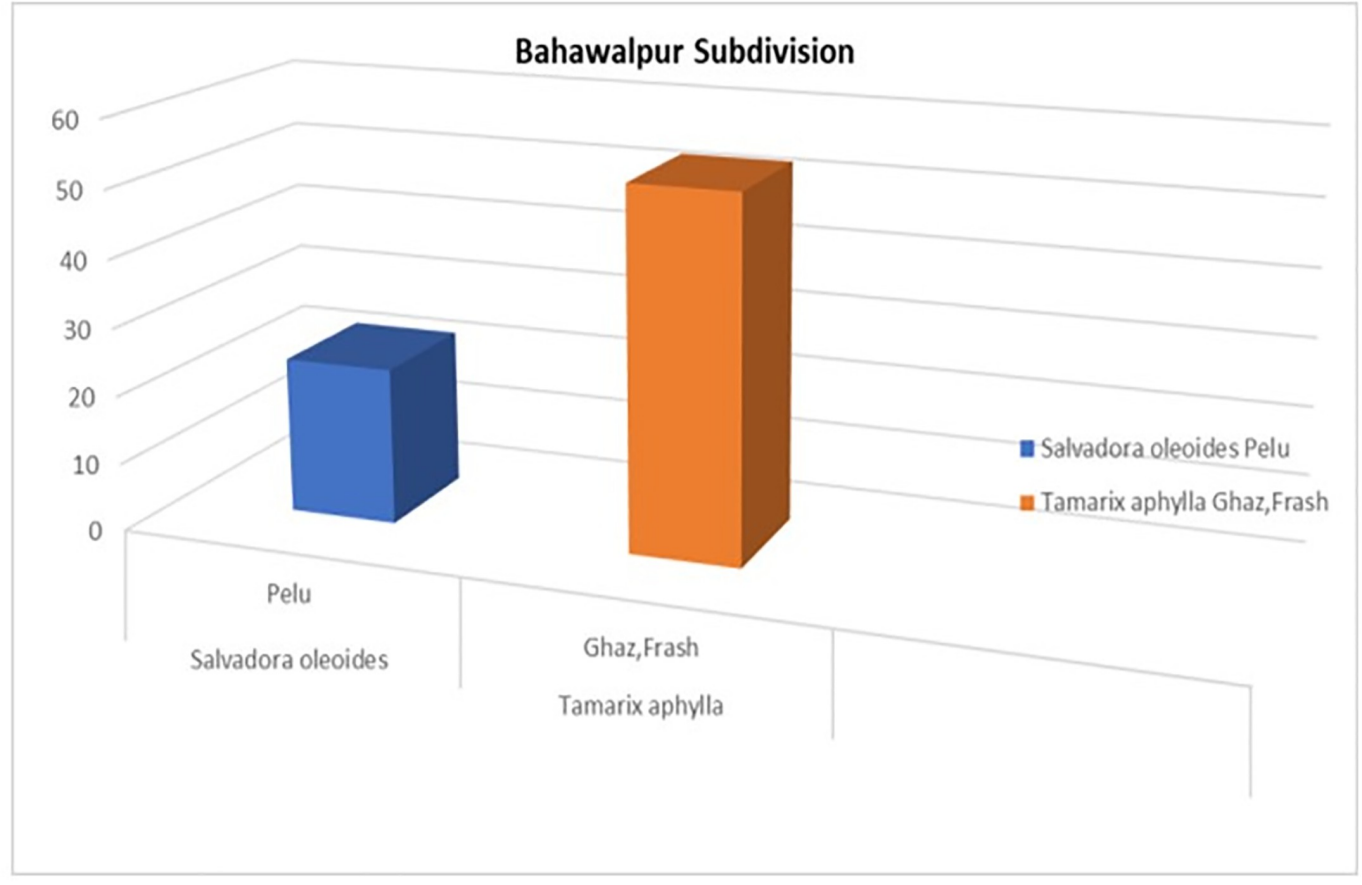
Conservation status of *Salvadora oleoides* and *Tamarix Aphylla*.

The tropical thorn forest in the Bahawalpur subdivision spans an extensive area of 83,187,303 square meters. During the survey, researchers visited the study area and randomly selected 20 plots, each with a radius of 17.84 meters, collectively covering an area of 0.1 square kilometers, as depicted in Fig 8. This sampling effort represents 0.01% of the total forest area [29]. Human activities, including habitat loss, exploitation, and slow species regeneration, are the primary drivers behind the decline of *S*. *oleoides* and *T*. *aphylla* populations. The survey revealed that *S*. *oleoides* exhibits a rare distribution pattern, with only 21 mature individuals scattered across the Bahawalpur subdivision. This species is often found in association with other plants, particularly in graveyards where human interference is minimal. The decline of *S*. *oleoides* is attributed to several factors: its use for medicinal purposes, fuelwood, and furniture, as well as the impacts of climate change and habitat loss. These pressures have significantly reduced its population, highlighting the urgent need for conservation efforts to protect and restore this species within its natural habitat.

## 4. Discussion

Human activities such as deforestation, pollution, and hunting have significantly contributed to the extinction of numerous species. The introduction of non-native species can further threaten indigenous flora and fauna. These factors have collectively led to the decline of many species. Despite the forest’s substantial ecological and socioeconomic importance, there have been limited efforts to map its distribution and implement protective measures. To effectively plan conservation strategies, it is crucial to identify which species are most at risk of extinction and their specific locations. This knowledge enables conservationists to prioritize their efforts and allocate resources to areas where endangered species are present.

Habitat suitability modeling was employed to map the current distribution of *S*. *oleoides* and *T*. *aphylla* in the Bahawalpur subdivision. These species were chosen due to their significant ecological roles in thorny ecosystems, their conservation status, and their suitability for habitat modeling. By focusing on these species, the study aims to enhance the understanding and conservation of biodiversity in arid ecosystems, thereby supporting sustainable management and protection of natural resources in the Bahawalpur subdivision. The model’s accuracy, measured by ROC-AUC, was greater than 0.976 for S. oleoides and 0.987 for T. aphylla. An AUC greater than 0.90 is considered excellent, indicating high model performance [30].

Recent investigations have focused on finding suitable habitats for various species, utilizing different methods and applications [31]. However, tropical thorn tree species have received relatively little research attention in Pakistan, primarily due to the lack of detailed forest inventory data, financial constraints, and the complexity of forest ecosystems [32]. Our findings align with previous studies that analyzed the distribution of Alternanthera philoxeroides in tropical regions using MaxEnt software and data from 570 samples collected between 2010 and 2017 [19, 21, 32]. Other researchers have focused on *T*. *aphylla* to determine its optimal habitat conditions, using a global climate change framework to predict its distribution. Satellite imagery, although valuable, is a costly approach in this assessment process [33].

Seasonal phenology and bioclimatic factors are crucial for future predictions [34]. Geospatial images are vital for studying species distribution as they provide insights into ecosystem health, functionality, and structure, as well as the underlying processes affecting local distributions [34]. Comprehensive geographic assessments and frequent data collection are essential for accurate analysis. Integrating satellite-based phenology data with bioclimatic parameters can enhance species occurrence predictions [35]. The MaxEnt model, requiring at least 100 samples for optimal results, demonstrated the highest prediction accuracy and capability in this study [36].

Historically, the rapid removal of popular trees from tropical thorn forests, driven by the demand for fuel from railways and steamers, led to a consistent decline in forested areas. The situation in Punjab highlights the urgent need to preserve the native flora of these forests, which are now at an alarming stage. This research analyzed the regional conservation status of two tropical thorn tree species against IUCN criteria (2001), finding *S*. *oleoides* to be endangered and *T*. *aphylla* to be vulnerable [37].

To ensure plant and seed production for forest operations, it is recommended to develop management strategies alongside the implementation of in situ conservation programs, such as the establishment of nurseries. The local population’s heavy reliance on plants for various needs has driven several species to the brink of extinction [38]. The rising demand for food, medicinal purposes, and firewood has been significant contributors to this issue. Overgrazing, smuggling, converting forests for cultivation, illegal tree cutting, and habitat loss exert immense pressure on the ecosystem, leading to environmental degradation [39].

Our research highlights the biological needs of these species and potential conservation strategies by determining their distribution patterns and habitat preferences. Conservation efforts should focus on protecting and regenerating habitats that support viable populations of *S*. *oleoides* and *T*. *aphylla*, especially in areas with high species diversity and habitat connectivity.The findings underscore the importance of conducting habitat suitability assessments for *S*. *oleoides* and *T*. *aphylla* to aid in their conservation. Using MaxEnt modeling, the research predicted the potential distribution of these species, identifying varying levels of suitability in the tropical thorn forests of the Bahawalpur subdivision. Remarkable accuracy was achieved, with ROC AUC values of 0.976 for *S*. *oleoides* and 0.987 for *T*. *aphylla*. The uneven distribution patterns of both species highlight the need for comprehensive conservation approaches to ensure their long-term survival. These results emphasize the crucial importance of preserving habitats and implementing effective management strategies to mitigate habitat degradation and enhance species resilience in the face of environmental challenges.

## 5. Conclusions

Our study revealed that both *S*. *oleoides* and *T*. *aphylla* are distributed throughout the tropical thorn forest of the Bahawalpur subdivision. Suitable habitats for these species ranged from low to highly favorable sites. *S*. *oleoides* is classified as endangered, while *T*. *aphylla* is considered vulnerable. Protecting these species requires enhanced collaboration and information sharing among plant scientists, as well as a collective effort to gather comprehensive data. Research on the spatial distribution and habitat suitability of *S*. *oleoides* and *T*. *aphylla* is a crucial step in their conservation, as it helps identify areas in need of protection. Moreover, organizing flowering events in the tropical thorn forest of Bahawalpur could highlight the importance of these species and promote their conservation.

Due to scarce resources and limited awareness of endangered plant species, valuable conservation opportunities have been missed. We recommend conducting more extensive analyses of climate change impacts on the Tropical Thorn regions. Raising public awareness about the consequences of unchecked climate change is also essential. We urge the government and local authorities to effectively detect climate change impacts and implement better mitigation policies. Grazing paths in open forests and revenue lands should be clearly identified and studied using scientific methods. Additionally, integrating GIS and remote sensing technologies into university curricula and promoting interdisciplinary collaboration between departments and universities can significantly enhance conservation efforts.
